# Application of chitosan/alginate nanoparticle in oral drug delivery systems: prospects and challenges

**DOI:** 10.1080/10717544.2022.2058646

**Published:** 2022-04-06

**Authors:** Shangyong Li, Hui Zhang, Kaiwei Chen, Mengfei Jin, Son Hai Vu, Samil Jung, Ningning He, Zhou Zheng, Myeong-Sok Lee

**Affiliations:** a School of Basic Medicine, Qingdao Medical College, Qingdao University, Qingdao, China; b Molecular Cancer Biology Laboratory, Cellular Heterogeneity Research Center, Department of Biosystem, Sookmyung Women’s University, Seoul, Korea; c Institute of Applied Sciences, Ho Chi Minh City University of Technology HUTECH, Ho Chi Minh City, Viet Nam; d Key Laboratory of Marine Eco-Environmental Science and Technology, First Institute of Oceanography, Ministry of Natural Resource, Qingdao, China

**Keywords:** Oral drug delivery, chitosan/alginate nanoparticle, insulin, ulcerative colitis, colon cancer

## Abstract

Oral drug delivery systems (ODDSs) have various advantages of simple operation and few side effects. ODDSs are highly desirable for colon-targeted therapy (e.g. ulcerative colitis and colorectal cancer), as they improve therapeutic efficiency and reduce systemic toxicity. Chitosan/alginate nanoparticles (CANPs) show strong electrostatic interaction between the carboxyl group of alginates and the amino group of chitosan which leads to shrinkage and gel formation at low pH, thereby protecting the drugs from the gastrointestinal tract (GIT) and aggressive gastric environment. Meanwhile, CANPs as biocompatible polymer, show intestinal mucosal adhesion, which could extend the retention time of drugs on inflammatory sites. Recently, CANPs have attracted increasing interest as colon-targeted oral drug delivery system for intestinal diseases. The purpose of this review is to summarize the application and treatment of CANPs in intestinal diseases and insulin delivery. And then provide a future perspective of the potential and development direction of CANPs as colon-targeted ODDSs.

## Introduction

Oral drug delivery systems (ODDSs) are effective strategy for intestinal disease treatment due to its convenient, patient-friendly, painless, and noninvasive properties (Renukuntla et al., 2013). Despite possessing numerous advantages, ODDSs are susceptible to absorption denaturation and degradation by the gastrointestinal tract (GIT) barriers, resulting in malabsorption and reduced effectiveness (even ineffective) at the disease site (Dos Santos et al., 2021). Meanwhile, protein and peptide drugs in ODDSs administration are susceptible to denaturation and degradation by digestive enzymes in the GIT (Haddadzadegan et al., 2022).

**Figure 1.**
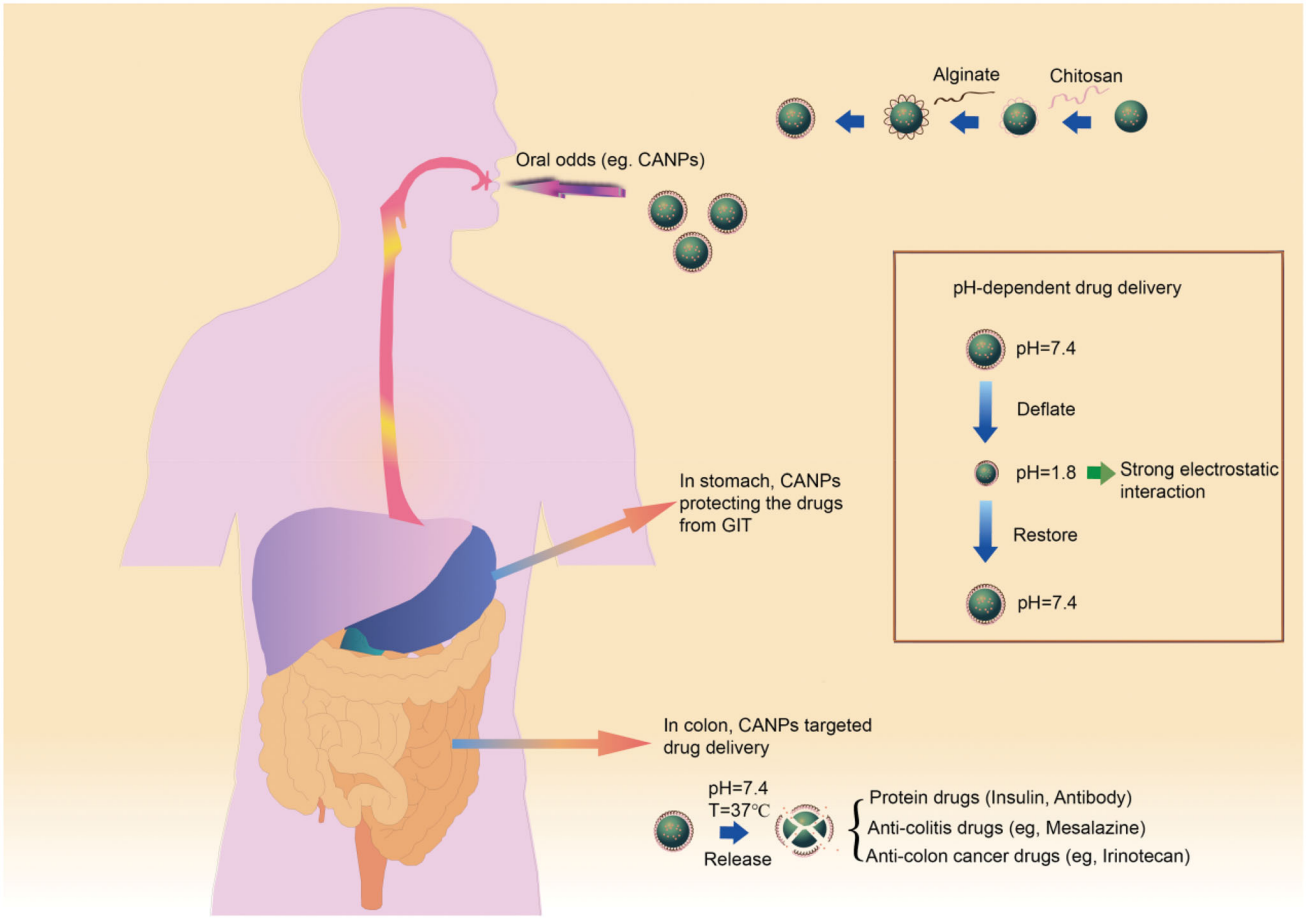
CANPs and its oral pH-dependent drug delivery property.

In general, the GIT is divided into three major regions: the stomach (pH 1.5–3.5), the small intestine (pH 5.5–6.8) and the large intestine (pH 7.0–8.0) (Chambin et al., 2006). Generally, oral colon-targeted drug delivery systems are pH-dependent and designed based on progressively increasing pH gradient in the GIT (Figure 1) (Keppler and Humpf, 2005). Typical pH-dependent nanomaterials include acrylate copolymers (eudragit), o-phenylene dimethyl acetate cellulose, chitosan, pectin, polylactic acid-hydroxyacetic acid copolymer (PLGA), etc. Among them, the polysaccharide-based materials offer significant advantages in terms of biodegradability, biocompatibility, adhesion, safety, and gelation properties (Yu et al., 2019).

Among those polysaccharides, marine derived polysaccharides have various advantages of being widely distributed, abundant content, simple to manufacture, biodegradable and biocompatible (Manivasagan et al., 2016; Chen et al., 2020). Chitosan and alginate as abundant and versatile marine polysaccharides, showed excellent mucosal adhesion, biodegradability and biocompatibility (Bhunchu and Rojsitthisak, 2014; Chu and Wang, 2020; Sudhakar et al., 2020; Taemeh et al., 2020). Chitosan/alginate nanoparticles (CANPs), prepared by the ionic gel method using chitosan and alginate as shells, have attracted attention as oral drug carriers (Hagras et al., 2022; Li et al., 2022). CANPs exhibited strong electrostatic interactions between carboxyl groups of alginate and amino groups of chitosan, resulting in shrinkage and gel formation at low pH, thereby protecting the drugs, from GIT and the aggressive gastric environment. Moreover, oral CANPs used for colon-targeted drug delivery have certain advantages, including increased drug absorption, improved oral drug bioavailability and therapeutic efficiency, reduced systemic toxicity and reduced administered dosage (Kumar and Mishra, 2008). These properties make CANPs an emerging and promising ODDSs.

The purpose of this review is to introduce the preparation methods and related properties of CANPs, and summarize their recent application on insulin delivery and intestinal diseases, e.g. colorectal cancer (CRC) and ulcerative colitis (UC). Furthermore, we also discussed the prospects and challenges of CANPs.

## Preparation methods and properties of CANPs

Ionic gel is the most commonly used methods for preparing CANPs, which was first proposed by Calvo et al. (Calvo et al., 1997). The mechanism of ionic gel method is based on electrostatic interactions between chitosan and alginate. Concretely, the physical interactions are from the negatively charged carboxylic acid group of alginate and the positively charged amino group of chitosan, which leads to shrinkage and gel formation at low pH, thereby protecting the drugs from GIT and the aggressive gastric environment (Lanjhiyana et al., 2013; Shinde et al., 2014). This method is generally carried out by dropping the drug-laden core polymer into an aqueous solution of polyvalent cations, followed by the addition of the polyanionic or anionic polymer under mechanical stirring at room temperature (Naskar et al., 2019; Lang et al., 2020).

Ionic gel method for CANPs has many advantages, such as the protocol are simple and fast, and easy to reach high encapsulation rate. Nevertheless, this method does not allow for a narrow particle size distribution, which will indirectly affect the drug loading capacity (Li et al., 2019).There are also other methods for CANPs preparation, including emulsification (Mao et al., 2016; Kyzioł et al., 2017; Khorshidian et al., 2019; Yue et al., 2020), electrostatic gelation (Aluani et al., 2017; Yoncheva et al., 2021), extrusion coagulation (ALQuadeib et al., 2020), co-precipitation (Yu et al., 2019), etcs. These methods are more complex than ionic gel method, while the control of particle size and distribution are more rigorous.

The properties of CANPs meet the criteria for an oral drug carrier. (1) CANPs protect the drugs in the GIT. CANPs as pH-dependent drug carriers could shrink at low pH through strong electrostatic interaction, thus protecting the drug from the aggressive gastric environment (Chen et al., 2019). (2) The size, shape and distribution of CANPs meet the requirements of ODDS. Significantly, the size of CANPs suitable for oral supplement in previous studies commonly ranges from 100-900 nm, which always exhibited higher efficiency of cellular internalization, stronger *in vitro* anti-inflammatory ability and better therapeutic effects. (3) CANPs are characterized by high drug loading capacity and high encapsulation efficiency. The method of preparation and the physicochemical properties of the drug determine the drug loading capacity and encapsulation rate of the carrier. (4) CANPs have suitable-sustained release properties. CANPs have better sustained release properties than naked drugs due to their pH-dependent nature, resulting in prolonged inflammatory or tumor suppression. (5) CANPs are nontoxic and degradable drug carriers. Alginate is a water-soluble, naturally occurring linear polysaccharide, and this biopolymer is nontoxic, biocompatible, biodegradable, with low immunogenicity and good mucosal adhesion, facilitating its use in oral drug delivery (Mukhopadhyay et al., 2015). Similarly, chitosan is biocompatible, biodegradable, nontoxic and non-immunogenic polymer with significant adsorption and antifungal activity, making it a great potential for use in food and pharmaceutical applications. Overall, CANPs have a wide range of applications as carriers for oral drug delivery (Venkatesan et al., 2017).

## Application of CANPs in colitis therapy

UC is a chronic disease that is increasing in incidence and prevalence every year (Segal et al., 2021) and is characterized by a long, incurable and recurrent course, with clinical manifestations such as bloody diarrhea, frequency, abdominal pain, weakness and fecal incontinence (Kobayashi et al., 2020). Oral drugs for UC treatment, such as 5-aminosalicylic acid (5-ASA) and glucocorticoid hormones (GCS), are rapidly and widely absorbed in the upper GIT, and may cause a range of side effects such as diarrhea, nausea, abdominal pain, headache, vomiting, and rash (Nunthanid et al., 2008). CANPs, as an emerging and promising ODDSs, have been reported for the treatment of UC (Table 1).

**Table 1.** Application of chitosan/alginate nanocarriers in the treatment of colitis.

Innovation points	Advantage	Particle size (nm)	Content (%)	Encapsulation efficiency (%)	Ref
PH-dependent release of curcumin from CAP1AG4 CH5@CUNCs	Enhanced accumulation in the inflamed colonic tissues	421 ± 14	20–80	90	(Oshi et al., 2020)
CD-Cur-CANPs with pH-sensitive and α-amylase-responsive release character-istics	Strong colonic biodistribution and accumulation, rapid macrophage uptake, promoted colonic epithelial barrier integrity and modulated production of inflammatory cytokines	462.1	3.49	88.89	(Li et al., 2021)
A pH-sensitive hydrogel formed by chitosan and sodium alginate under the mechanochemical force	5-ASA colon-targeted delivery system without a crosslinking agent	489.57 ± 118.07	3.77	–	(Xu et al., 2021)
Novel pH-sensitive hydrogel beads based on gelatin/sodium alginate/ chitosan loaded with propolis ethanolic extracts	Synthesis of pH-sensitive hydrogel beads for different propolis ethanolic extracts	10^6 ^−2 × 10^6^	–	–	(Ceylan et al., 2021)
A series of curcumin loaded polymeric nanoparticle (NPs) with different particle sizes	The surface functionalization of PF127 can enable NPs to penetrate through the mucus layer Improve the cellular uptake efficiency of NPs by macrophages	185–884	5.1–6.1	73.2–89.6	(Zhou et al., 2020)

Oshi et al. developed orally administered core-shell nanoparticles with a core of curcumin nanocrystals (CUNCs) and a shell layer of CANPs to successfully reduce symptoms associated with inflammation in dextran sodium sulfate (DSS)-induced colitis mice model (Oshi et al., 2020). Through this ODDS, they aimed to precisely deliver CUNCs to the colon and protect the drugs from GIT, resulting in a significantly therapeutic effect. During UC treatment, pH-triggered surface charge inversion of CANPs promotes the adhesion and accumulation of CUNCs in inflamed colonic tissue by causing them to interact with negatively charged mucins in the mucosa.

In our previous study, an effective enzyme-triggered controlled release system using a curcumin-cyclodextrin (CD-Cur) inclusion as the core and CANPs as the shell (Li et al., 2021). This specific colon-targeted drug delivery system showed significant pH-dependent properties, protecting the drugs through GIT. Meanwhile, taking advantage of the characteristics of α-amylase produced by gut microbiota to specifically hydrolyze the CD-Cur core, this enzyme-triggered controlled release system showed promising therapeutic effects on DSS-induced colitis in mice, well protected the colon, promoted the integrity of the colonic epithelial barrier and remodeled the intestinal microbiota.

In order to overcome the problems of complex preparation process, poor biocompatibility and delayed drug release of current oral targeted drug delivery systems, Xu et al. prepared 5-ASA-loaded colon-targeted hydrogels without non-crosslinkers in a single step by applying pH-sensitive characteristics through a mechanochemical approach (Xu et al., 2021). Experimental studies have shown that this colon-targeted hydrogel has a release rate of 28.19% in simulated gastric fluid, which is 62.83% lower than the 5-ASA active pharmaceutical ingredient (API), while the release rate in simulated colonic fluid is similar to that of the 5-ASA API. It can be seen that colon-targeted drug delivery systems are simple to prepare, and can improve the therapeutic effect of drugs by targeting the lesion sites in the colon.

Propolis is a resinous substance with anti-inflammatory, antibacterial, and antioxidant properties, and ethanolic extracts are the main form of propolis used in the treatment of colitis. Ceylan et al. synthesized a novel pH-sensitive hydrogel bead based on gelatin/sodium alginate/chitosan (GEL/SA/CS) loaded with propolis ethanol extract (PE) (Ceylan et al., 2021). Propolis was distributed in the carrier matrix by using GEL in the formulation and the outer surface of the carrier beads was covered with CS to increase the antibacterial properties, as well as to control the rate of propolis release. These propolis hydrogel microbeads have been shown to increase antibacterial activity in gastrointestinal environments at different pH levels and over different time periods, better targeting colitis treatment.

Recent studies have indicated that particle size is a critical factor influencing the phagocytic response of macrophages (Anderson et al., 2015; Jo et al., 2015; Jindal, 2017). To elucidate the effect of particle size on the cellular internalization and anti-inflammatory activity of orally administered nanomedicines, Zhou et al. prepared CuR-loaded nanoparticles with hydrodynamic particle sizes of 185, 474 and 883 nm, named NPs (200), NPs (500) and NPs (900), respectively, using a single-emulsion solvent volatilization method (Zhou et al., 2020). *In vitro* cellular uptake experiments showed that NPs (900) had the highest phagocytic efficiency on macrophages, while *in vivo* experiments demonstrated that NPs (900) had better efficacy. It follows that, within a certain size range, larger nanoparticles seem to respond better to phagocytosis by macrophages and have better efficacy.

Several of the nanocarriers mentioned above have been optimized with different treatments so that the drugs can be more accurately targeted to the inflammatory sites of colitis to work. In summary, nanoparticle systems have been identified as promising strategies for colon-targeted drug delivery purposes (Naeem et al., 2020). On the other hand, we propose that when treating UC via nanocarriers, consideration should be given to including physiological or pathological conditions related to cells/tissues damaged, loss of barrier function, mucus damage and changes in microbiota homeostasis, based on which to design more complex drug delivery system.

## Application of CANPs in Colon cancer treatment

CRC is the third most common malignancy and the fourth leading cause of cancer-related death in the world. Until now, surgical resection is the most common treatment for CRC, but it is usually accompanied by the removal of a portion of a healthy colon or rectum and nearby lymph nodes. Even worse, a large number of CRC patients have common complications during surgery and often develop recurrence, which makes CRC difficult to cure. Common chemotherapeutic drugs used to treat CRC are 5-fluorouracil (5-FU), oxaliplatin, and methotrexate, which are usually administered intravenously, exposing healthy tissues to toxic chemotherapeutic drugs, which limit the dose and effectiveness of the drugs and have more side effects. CANPs, as a colon-targeted drug delivery system, have become a potential CRC drug nanocarrier (Table 2).

**Table 2.** Application of chitosan/alginate nanocarriers in the treatment of colon cancer.

Innovation points	Advantage	Particle size (μm)	Content (%)	Encapsulation efficiency (%)	Ref
Alginate and alginate-chitosan beads containing celecoxib solubilized into a self-emulsifying phase	Delay the drug release in acidic environment and to promote it in the intestinal compartment	715–896	39.78–49.63	–	(Segale et al., 2016)
Efficacy of the novel biopolymeric complex multiparticulate system consisting of chitosan, succinate and alginate for the capecitabine	CS-SA beads prolong the release of capecitabine in the colonic region, and also enhance antitumor efficacy	846.21 ± 5.46	98.26 ± 3.14	85.63 ± 2.03	(Sinha et al., 2018)
5-Fluorouracil (5-FU) loaded layer-by-layer (LBL) film prepared by sequential adsorption of chitosan and alginate polyelectrolytes	The drug payload could be increased by preparing films in a LBL self-assembled manner	145.0 ± 11.6	1837.2 ± 119.9 (μg/cm^2^ area of film)	–	(Janardhanam et al., 2020)
Optimized chitosan-Ca-alginate beads loaded with acid-resistant particles of 5-FU	Prolong residence time in colon, control release of encapsulated drug	14.74 ± 0.09	49.01 ± 1.8 (mg/g MP)	72.78 ± 1.10	(Glavas Dodov et al., 2009)
Crosslinked polycation MPs loaded with acid-resistant particles of 5-FU and functionalized with wheat germ agglutinin (WGA)	Deliver the drug molecules to colon region, affect the transport of 5-FU into the cells	14.7	82–90	72.8	(Glavas-Dodov et al., 2013)
Sodium alginate as the shell layer and the quercetin-loaded chitosan nanoparticles and prebiotics as the core layer	Prebiotic activity and an enhanced colon cancer prevention property	188.3 (nm)	11.53	92.2	(Wen et al., 2018)
Innovation points: Alginate-coupled trimethyl chitosan (TMC) loaded anti-gp130 and anti-S1PR1 siRNAs	Immunotherapy of cancer by specific silencing of tumor target antigens by NPs loaded with small interfering RNA (siRNA)	110 (nm)	30	–	(Rostami et al., 2020)

Celecoxib is a non-steroidal anti-inflammatory drug which has significant anti-cancer activity against CRC. Lorena Segale et al. used CANPs to prepare celecoxib microspheres which can minimize the release of celecoxib in the acidic environment and enhance its release in the intestinal lumen (Segale et al., 2016). Furthermore, they demonstrated that CANPs beads could ensure colonic administration of celecoxib by reducing the drug release rate under neutral pH conditions. Sinha et al. developed a novel biopolymeric complex multiarticulate system consisting of chitosan succinate and alginate (CS-SA) to encase capecitabine for CRC therapy. First, they optimized CS-SA using a Box-Behnken design according to three considerations of encapsulation efficiency, size, and release efficiency. The experiment data showed that the optimized CS-SA swelled the most in the intestinal environment of pH 7.4, while hardly swelled in the acidic environment, thus achieving the effect of protecting the drug and effectively acting on the colon (Sinha et al., 2018).

Layer-by-layer (LBL) self-assembled methods was also used to prepare CANPs for oral CRC treatment (Janardhanam et al., 2020). This LBL self-assembly method prepares films that greatly improve the high loading efficiency of 5-FU by using polycaprolactone (PCL, 95% w/w) as the backing. In addition, selective tumor lesion binding and targeting capabilities were obtained by functionalizing the outer-most layer with folic acid. It was found that the 5-FU LBL film not only had higher stability, but also exhibited a stronger cytotoxic effect on colon cancer cell lines than the 5-FU solution preparation.

Furthermore, to improve the oral administration ability of 5-FU in CANPs, Dodov et al. prepared lectin-coupled chitosan-Ca-alginate microparticles (MPs) (Glavas-Dodov et al., 2013). The lectin used herein was wheat germ agglutinin (WGA, 36 kDa), one of the least immunogenic lectins that has been shown to be more specific to human intestinal cell lines and human colon cells (Glavas Dodov et al., 2009). The experimental results showed that MPs loaded with 5-FU acid-resistant particles could deliver 5-FU to the colon area after being functionalized by WGA, and had good interaction with the colonic mucosa surface and could prolong the action time in the colon area.

Quercetin is a natural flavonoid compound that has the effect of preventing colon cancer, however, poor stability and low oral bioavailability limit its use. Wen et al. prepare quercetin loaded CANPs, using sodium alginate as shell, quercetin-chitosan nanoparticle and as the core layer. Then, they prepared a quercetin-loaded electrospunfiber mat (Q-EFM) containing prebiotics (GOS) (Wen et al., 2018). The experimental results showed that quercetin could be sustained and targeted released in the colon by Q-EFM, and the presence of GOS could increase the quercetin release rate. Therefore, this Q-EFM with prebiotic function can be used as a new type of colon-targeted delivery system to enhance anti-CRC effect.

An interconnected network between S1P/phosphosynuclein-1-phosphate receptor 1 (S1PR1), IL-6/glycoprotein 130 (GP130), and signal transducer and activator of transcription 3 (STAT3) signaling pathways, promotes cancer progression. Therefore, Rostami et al. used alginate coupled trimethyl chitosan (ATMC) NPs loaded with small interfering RNA (siRNA) to silence STAT3 upstream targets, including S1PR1 and GP130, finding that the siRNA-loaded NPs successfully inhibited the colony formation and migration of cancer cells (Rostami et al., 2020).

An important issue for drug delivery systems for the treatment of CRC is that the tumor-forming microenvironment leads to tissue hypoxia, which releases a protein called hypoxia-inducible transcription factor (You et al., 2016), the abnormal release of this protein will ultimately lead to vascular damage, and the damaged blood vessels will then hinder the delivery of essential nutrients to the diseased tissue, directly affecting the absorption of drugs, which puts forward higher requirements for the efficiency of the drug delivery system. Furthermore, drug delivery must pass through a kinetic barrier from blood flow to intracellular transport, which may be compromised in tumor tissue due to the heterogeneous structure of the tumor vessels, thus impairing the homogeneity of drug distribution (Kato et al., 2013; Suarato et al., 2016). Therefore, when designing colon-targeted drug delivery systems, the above-mentioned problems associated with CRC should be fully considered, especially the physiological and pathological characteristics associated with CRC.

## The application of CANPs in oral insulin delivery

Diabetes is one of the most common chronic metabolic diseases in the world. Repeated subcutaneous injections of insulin disturbs the lives of patients with insulin-dependent diabetes. Oral administration of insulin is patient-friendly and cost-effective. As a protein drug, the orally administered insulin must pass through many physiological barriers, such as GIT, mucus layer, intestinal epithelium, then finally reach the circulatory system (Chen et al., 2011). Thus far, oral absorption of insulin remains a major scientific challenge. First, oral insulin must be effectively transported along the GIT tract without being degraded by the acidic conditions in the stomach and proteases in the gastrointestinal tract. Secondly, insulin is a hydrophilic protein, which is difficult to be encapsulated in a hydrophobic macromolecular carrier. Third, the bioavailability of untreated insulin is extremely low due to the first-pass effect in the liver. Thus far, many researchers have used CANPs as the carrier of insulin delivery (Table 3).

**Table 3.** Innovation of chitosan/alginate nanoparticles in the treatment of diabetes.

Innovation points	Advantage	Particle size (nm)	Content (%)	Encapsulation efficiency (%)	Ref
Calcium ions were added into chitosan/ alginate nanoparticles to form microspheres	Protect insulin	194.25 ± 51.25	11.45	23.70	(Li et al., 2021)
Stearic acid and alginate form alginate stearic acid nanopartic-les (ASAN), then cross-linked with oleic acid modified chitosan	Improve the encapsulation of insulin	618.87 ± 6.57	6.44	76.69	(Alfatama et al., 2018)
Alginate combined with C-18 to form alginate-c18 conjugate nanoparticles (AC18N), then cross-linked with oleic acid modified chitosan	Reduce its toxicity and enhance mucus penetration and intracellular transport	522.50 ± 66.47	3.77	44.87	(Alfatama et al., 2018)
Polyalacturonic acid (PGLA), chitosan, and alginate NPs	Avoid intestinal degradation caused by pH sensitivity and improves the overall blood sugar lowering effect	225 ± 75	34.13	35.56	(Zhang et al., 2018)
Bichitosan/albumin coated alginate /dextran sulfate nanoparticles	Improve mucosal adhesion efficiency and insulin permeability	313.2 ± 2.8	10.10	72.40	(Lopes et al., 2016)
Calcium phosphate is the core of nanoparticles, Vitamin B12 grafted Chitosan and sodium alginate used as cationic and anionic polyelectrolyte, respectively.	Enhance NPs uptake	212.6 ± 6.38	7.83	75.16	(Verma et al., 2016)

Additionally, transit time is another factor that affects the oral delivery and bioavailability of drugs to the colon. The normal transit time in the small intestine is approximately 4 h, with an inter-individual variability of 2 to 6 h; that of colon, however, is relatively variable, ranging from 6 to 70 h.

CANPs as a pH-sensitive ODDSs that protect insulin from damage and degradation in low pH environments, as well as gastrointestinal enzymes. Chen et al. used CANPs as carriers for the Cp1-11 peptide/insulin complex to protect insulin from damage and to enhance the biological activity of monomeric insulin (Chen et al., 2019). Their results indicate that encapsulation in CANPs is an effective way to protect the complex of insulin/Cp1-11 peptide. CANPs thus are one of the promising tactics to deliver insulin to treat type 1 diabetes patients. In another context, Alfatama et al. used alginate-C18 conjugated NPs loaded in TPP-linked chitosan oleic acid to improve mucus penetration and intracellular transport and to compensate for the shortcomings of alginate gels with high porosity and rapid drug release (Alfatama et al., 2018).

The core-shell structure is the most commonly used form of CANPs. Zhang et al. developed a core-shell CANPs based on positively charged chitosan and negatively charged alginate to functionally simulate the neutral surface of the virus formed through a highly negatively charged surface to increase the mucus permeability of the insulin-loaded nanocomplex (Zhang et al., 2018). The negatively charged alginate coating reduced the mucus penetration barrier of the nanocomposite and increased villi absorption and intestinal penetration. Bichitosan/albumin coated alginate-DSS-NPs further improved the mucosal adhesion efficiency and insulin permeability, so as to enhance the oral administration of insulin (Lopes et al., 2016). Approximately 70% of insulin was successfully retained in NPs in the gastric environment and then slowly released upon entry into the intestinal environments.

LBL coated CANPs can also be used for insulin protection. Zhang et al. developed an LBL-based oral insulin delivery system which is automatically formed by the ionic attraction between polygalacturonic acid (PGLA), chitosan, and alginate. The combination of particles avoided intestinal degradation caused by pH sensitivity and improved the overall blood sugar lowering effect (Zhang et al., 2018). Vitamin B12 was combined in LBL coated CANPs to improve the stability of insulin against gastric enzymes, and control insulin release to reach its physiological concentration (Verma et al., 2016). Li et al., prepared chitosan and TPP into NPs, and then further coated the surface of the nanoparticles with alginic acid and added calcium ions to form microbeads to protect insulin (Li et al., 2021). Such NPs showed excellent controlled-release properties in simulated stomach and small intestine solutions. The insulin-loaded CANPs were even capable of controlling hyperglycemia within 100 hours, which offers a potential therapeutic strategy for the treatment of diabetes.

Although some progress has been made over the years with oral insulin carriers, this work still requires further research before it can be truly used in the clinic. As mentioned above, the oral insulin delivery systems based on CANPs can achieve protection and transport of insulin and improve the physicochemical and biological stability and loading efficiency of insulin (Li et al., 2013). However, the utilization of oral insulin is still too low compared to intravenous subcutaneous injection. In addition, to maintain the stability of insulin in the gastrointestinal tract is also a crucial issue, especially for those oral delivery systems that use mucus adhesion systems to allow for extended insulin residence time (Wong, 2010). We believe that an ideal oral insulin delivery system should be biocompatible and biodegradable, and be able to remain stable in the gastrointestinal tract and achieve appropriate bioavailability.

## Prospects and challenges

Application of colon-targeted oral drug delivery is unarguably the most convenient approach for the treatment of common intestinal diseases such as UC and CRC (Ling et al., 2019). The oral therapy of UC and CRC is a challenging task mainly due to its high complexity. There have been several research efforts in the field of colon-targeted drug delivery systems for the treatment of UC and CRC with varying degrees of success. Nonetheless, the effectiveness is significantly reduced due to enzymatic digestion in the stomach, make many challenges to efficiently deliver drugs to the colon (Yu et al., 2009). CANPs showed strong electrostatic interaction between alginate and chitosan, which leads to shrinkage and gel formation at low pH (in stomach), thereby protecting the drugs from GIT and the aggressive gastric environment (Ensign et al., 2012). Colon-targeted drug delivery of CANPs in UC and CRC have certain advantages, such as increased drug absorption, improved oral drug bioavailability and therapeutic efficiency, reduced systemic toxicity and reduced administered doses (Dos Santos et al., 2021).

The major obstacle for oral drug delivery via nanocarrier is the fact that it needs to cross several natural barriers of the human body before the incorporated drug can reach the target cell. Once ingested, CANPs will protect the drug from the acidic environment and proteolytic ‘thunderstorms’ in the stomach. After leaving the stomach, drug loaded CANPs enter the small intestine and are transported along the duodenum, jejunum, and ileum. The pH-dependence determines the drug loading and release rate of CANPs in colon. To improve the pH-dependence of CANPs material, Zhang et al. developed an automatic oral insulin delivery system formed by the ionic attraction between PGLA, chitosan and alginate which made CANPs more effective against gastric acid erosion *in vitro* experiments (Zhang et al., 2018). In addition, the report of enhancing the stability of CANPs in acidic environments by adding gelatin was also mentioned previously (Ceylan et al., 2021). Therefore, exploring more effective and nontoxic pH-stable materials is an important direction for CANPs as colon-specific therapies.

The current researches about colon-targeted delivery of CANPs have focused more on how to avoid the degradation of gastric acid, especially the pH-dependent release *in vitro*. However, after the drug enters the colon, it is still unclear whether it can play a normal pharmacological effect and be metabolized by the intestinal tract. 5-ASA, also known as mesalamine, is the treatment of choice for mild to moderate cases of UC. In our previous study, 5-ASA was encapsulated in microspheres prepared by CANPs-based hydrogels (Wu et al., 2021). This hydrogel microsphere had different swelling abilities in different pH media, which had colon-targeting and strong retention ability in colitis mice, and the microsphere was biodegradable, achieving a therapeutic effect superior to free drugs. However, whether this sustained-release system can overcome its extensive hepatic and intestinal metabolism, resulting in the formation of less active N-acetyl-5-ASA, has not been investigated. Thus, in addition to studying more effective oral drug delivery materials, more attention should be paid to the efficacy and absorption of loaded drugs in the colon in the future.

Moreover, some challenges remain in the clinical treatment of colon targeted drug delivery for intestinal disease. The location of this final GIT moiety presents a series of physical, chemical and enzymatic barriers that hinder the delivery and stability of oral drugs and is considered one of the major issues to overcome. At present, the application of CANPs is still at the verification of *in vitro* and animal experiments. CANPs still have a long way to go to overcome the several challenges facing oral therapeutic delivery and intestinal disease therapy.
